# Health Behavior Change Intervention Preferences Expressed by American Indian Cancer Survivors From a Southwest Tribal Community: Semistructured Interview Study

**DOI:** 10.2196/51669

**Published:** 2024-03-27

**Authors:** Jennifer Erdrich, Felina M Cordova-Marks, William O Carson, Jennifer W Bea, William R Montfort, Cynthia A Thomson

**Affiliations:** 1 Department of Surgery College of Medicine University of Arizona Tucson, AZ United States; 2 Department of Health Promotion Sciences Mel and Enid Zuckerman College of Public Health University of Arizona Tucson, AZ United States; 3 Department of Chemistry and Biochemistry University of Arizona Tucson, AZ United States

**Keywords:** Native American cancer disparities, diet, physical activity, prehabilitation, native, exercise, fitness, interviews, thematic analysis, lifestyle, Apache

## Abstract

**Background:**

While many factors, including social determinants of health, affect cancer mortality, one modifiable risk factor that may contribute to cancer disparities is obesity. The prevalence of obesity in the American Indian/Alaska Native population is 48.1% per the Centers for Disease Control and Prevention. The overall cancer mortality for the American Indian/Alaska Native population is 18% higher than the White population as reported by the American Cancer Society. Interventions tailored to American Indian/Alaska Native communities that promote healthy lifestyle behaviors after cancer diagnosis and prior to cancer surgery (prehab) might improve cancer outcomes for this population.

**Objective:**

The aim of the study is to characterize the lifestyle behaviors of San Carlos Apache cancer survivors and identify preferences for the adaption of a prehab intervention.

**Methods:**

Semistructured interviews and validated questionnaires were completed with San Carlos Apache cancer survivors (N=4), exploring their viewpoints on healthy lifestyle and cancer risk and preferences for program development. A thematic content analysis was conducted.

**Results:**

Participants had an average BMI of 31 kg/m^2^ and walked 53 minutes daily. The majority of participants reported a high willingness to change eating habits (n=3, 75%). All 4 reported willingness to participate in a diet and exercise program. Important themes and subthemes were identified: (1) cancer is perceived as a serious health condition in the community (N=4, 100%); (2) environmental exposures are perceived as cancer-causing threats (n=3, 75%); (3) healthy diet, exercise, and avoiding harmful substances are perceived as mitigating cancer risk (n=3, 75%); (4) barriers to healthy habits include distance to affordable groceries (n=3, 75%) and lack of transportation (n=2, 50%); (5) there is high interest in a prehab program geared toward patients with cancer (N=4, 100%); and (6) standard monitoring practiced in published prehab programs showed early acceptability with participants (N=4, 100%).

**Conclusions:**

Collaboration with tribal partners provided important insight that can help inform the adaptation of a culturally appropriate prehab program for San Carlos Apache patients diagnosed with cancer.

## Introduction

Cancer is the second leading cause of death for the American Indian/Alaska Native population with related disparities in cancer screening, incidence, stage at diagnosis, and timely treatment [1-8]. Consequences of colonialism such as geographic isolation, poverty, and social determinants of health have not only created steep disadvantages in the progression of cancer but also contributed to detrimental behaviors related to diet and exercise that place tribal members at higher risk for worse cancer outcomes [9]. There are 13 obesity-related cancer subtypes that account for 40% of all cancers diagnosed in the United States, which confers significant risk for the American Indian/Alaska Native population, given the 48.1% prevalence of obesity for this population [10,11]. Published data suggest that brief exercise interventions following diagnosis of solid tumor malignancies might lower cancer progression, specifically through the regulation of metabolism and the immune response [12-14]. Epidemiologic studies have suggested anti-inflammatory foods can improve cancer outcomes [15-18]. While anti-inflammatory diet and exercise trials are increasing for patients with cancer in the general population, few have involved patients from American Indian/Alaska Native populations [19].

The Partnership for Native American Cancer Prevention, established in 2002, is a program that strives to alleviate the unequal burden of cancer among Native Americans in the Southwest through research, training, and community outreach [20]. Through the Partnership for Native American Cancer Prevention, the investigators of this project partnered with the San Carlos Apache Healthcare Corporation (SCAHC) with approval from the Tribal Council. The San Carlos Apache reservation, established in 1871, spans 1.8 million acres of Sonoran Desert and alpine forests in Southeastern Arizona and is home to 10,815 people. The tribal government and SCAHC are committed to providing quality care that honors Apache traditions and promotes Apache values of well-being, goodness, balance, harmony, and beauty [21-23].

Lifestyle modifications in nutrition and exercise prior to oncologic treatment are key strategies of prehabilitation (prehab) and have been shown to optimize patients’ functional capacity and improve postoperative outcomes [24-26]. Results from this work are being used to propose the next evolution of this project, which is the adaptation of a culturally appropriate prehab program for San Carlos Apache patients diagnosed with cancer who are preparing for surgery. It was recognized that San Carlos Apache community members, specifically those who have experienced the cancer spectrum from diagnosis to survivorship would have the best insight for developing a program specifically tailored to the San Carlos Apache community. The purpose of this project was to conduct semistructured interviews and administer validated questionnaires to characterize lifestyle behaviors of San Carlos Apache cancer survivors and identify appropriate approaches for the adaption and delivery of a lifestyle intervention for San Carlos Apache patients diagnosed with cancer.

## Methods

### Study Design

The principles of community-based participatory research were followed throughout the study. SCAHC and Tribal Council members advised the project activities, which were conducted with their approval and collaboration. Qualitative semistructured interview questions were developed through an iterative process supervised by faculty experts in qualitative methods. The questions were then presented to tribal community members at SCAHC to ensure the content was appropriate. The questions reflected the content of published prehab programs and focused on understanding the most acceptable approach for program adaptation for San Carlos Apache patients. The questions were organized according to four areas: (1) knowledge of cancer and its causes and prevention, (2) perspectives on cancer and lifestyle, (3) diet and physical activity preferences, and (4) attitudes and beliefs regarding diet and physical activity interventions for survivors. In total, 32 semistructured interview questions were asked of participants, including potential subprompts. Interview data were complemented with facilitator administration of 2 validated questionnaires about nutrition and physical activity. These were the Rapid Eating Assessment for Participants-Shortened Version (REAPS) and the International Physical Activity Questionnaire (IPAQ), which were selected because of the validity, reliability, literacy, and low participant burden of these brief instruments [27-30].

Interviews were conducted by the principal investigator through password-protected Zoom (Zoom Video Communications). The participants accessed the platform through telephone dial-in. The camera feature was not used, names were avoided, and data were deidentified. The participants’ responses were audio-recorded with permission and transcribed. The interviewer took handwritten notes of the responses to cross-check the transcription and verify accurate audio recording. Minor errors were corrected on the final transcriptions.

An independent reviewer with expertise in qualitative methods and American Indian/Alaska Native health was consulted to perform a thematic content analysis of the deidentified transcripts using a matrix system grounded in a framework focused on determinants of health from an Indigenous perspective. This method has been used for analyzing qualitative data with other tribes and is described elsewhere [31]. Briefly, the matrix was used to organize the questions and responses into a coded summary chart, wherein similar responses were grouped together after an iterative process of grouping, regrouping, and refining. From this, the content was grouped into themes and subthemes. Direct quotes particularly illustrative of key themes were extracted. The reviewer summarized the principal findings for intervention planning.

### Participants and Recruitment

Tribal members who are cancer survivors residing on the San Carlos Apache reservation, aged at least 18 years, and diagnosed with cancer within the previous 15 years were recruited for semistructured interviews. Recruitment efforts were centered on the recommendations of the social workers and physicians at SCAHC. The social workers at SCAHC engage all patients diagnosed with cancer, not only at diagnosis but also during survivorship, per their facility’s routine. This was recognized to position the social workers in contact with the highest volume of potential participants, who were approached on a rolling basis over the course of 3 months. Furthermore, social worker engagement was intended to minimize influence since the social workers are neither part of the university study team nor the clinical care team. The social workers introduced the study to the survivors, and for those who expressed interest in the study, the social workers verified that prospective participants gave permission to share their contact information. Each prospective participant underwent a brief screening by the study lead (JE) to verify eligibility following a telephone screening script. If individuals agreed to participate, they were consented and then scheduled for a semistructured interview on a separate day.

### Ethical Considerations

This project was developed in collaboration with the San Carlos Apache Tribal Council and SCAHC. Human Subjects approval was obtained from the University of Arizona Institutional Review Board (study ID# CR00001226), SCAHC Institutional Review Board, and San Carlos Apache Tribal Council Health Committee. All materials were approved by the Tribal Council and SCAHC. The tribal support letter was submitted to the University of Arizona. SCAHC Institutional Review Board and Tribal Council approved the content of this paper and its submission. Informed consent was obtained from all participants (verbal or written option, all participants chose verbal and were sent a hard copy of the written consent form). Upon completion, participants received a gift card as compensation for their time. Privacy and confidentiality were upheld in the data collection, and the data were deidentified.

## Results

### Demographics and Questionnaires on Physical Activity and Diet Behaviors

A total of 4 participants were interviewed from May to July 2022. SCAHC referred 12 potential participants; 8 were successfully contacted and 4 agreed to study participation (response rate of 4 of 12 potential participants or 33%). Interviews lasted 60-80 minutes. The average age of participants was 64 years, with 1 male patient and 3 female patients. The participants were diagnosed with cancer between 2015 and 2021. A family history of cancer was reported by 3 (75%) participants. All were overweight or obese with an average BMI of 31 kg/m^2^. There were 2 (50%) participants who reported past smoking, and 1 (25%) who reported past moderate alcohol consumption. Based on the IPAQ, the participants reported an average of 158 minutes of vigorous activity daily, 90 minutes of moderate activity daily, and 225 minutes of sitting daily. All patients reported walking specifically as part of their physical activity routine and reported an average of 53 minutes of daily walking. Based on the REAPS, it was elicited that 2 (50%) participants ate 4 or more meals per week from restaurants or takeout, 3 (75%) ate 2 or more servings of fruit per day, 3 (75%) ate 2 or more servings of vegetables per day, 3 (75%) answered they usually or sometimes use processed meats, 2 (50%) regularly ate fried foods, 3 (75%) rarely ate sweets, and 2 (50%) reported drinking 16 ounces or more of nondiet soda or punch per day. On a scale of 1-5, for willingness to make changes in their eating habits where 1=very willing and 5=not at all willing, 3 (75%) participants answered positive willingness with a score of 1-2.

### Semistructured Interviews: Qualitative Analysis

#### Overview

The following themes were revealed (Textbox 1): (1) cancer as a serious health condition in the community, (2) environmental exposure and contamination as a main cause of cancer, (3) personal choices as risk factors or prevention, (4) barriers, (5) acceptability of diet and physical activity programs, and (6) acceptability of monitoring and biospecimen collection.

**Qualitative semistructured interview themes and subthemes.**

- Cancer as a serious health condition in the community
- Environmental contamination and exposure as a cause of cancer
  Occupational exposure
  Chemical in the community
  Water contamination
- Personal choices as risk factors or prevention
  The power of food, exercise, and lifestyle
- Barriers
  Distance and transportation
  The built environment
- Acceptability of diet and physical activity program
  Considerations for program development
    - Support and encouragement
    - Central location
    - Education
- Acceptability of monitoring and biospecimen collection

#### Cancer as a Serious Health Condition in the Community

All participants affirmatively stated that cancer is a problem for the San Carlos Apache community. Participants defined cancer as a serious condition with the words “horrible,” “deadly,” and “life-threatening.” Based on this perception, all 4 participate in cancer screening and surveillance. Half of the participants endorsed the concept of cancer as potentially curable and the importance of health care in cancer prevention.

#### Environmental Exposure and Contamination as a Main Cause of Cancer

There were 3 (75%) participants who described an environmental issue as a main cause of cancer.

##### Occupational Exposure

Exposure to harmful chemicals through occupation was described by 2 (50%) participants as a main cause of cancer:

Some of it is asbestos for a lot of our workers who worked in these asbestos mines.

Another connected cancer to exposures in the workplace (workplace redacted for identity protection):

I used to be a [occupation redacted]. It was the environment we worked in. That’s why we’re doing an early retirement, [workplace redacted], because of our environment, what we ingested from the environment.

##### Chemicals in the Community

A history of chemicals in the community believed to be linked to cancer was vividly described by 1 (25%) participant:

I remember I was very young when they sprayed our area. I remember the planes hanging out in our area and how it killed a lot of our trees. I remember the one thing that really impacted me was the poppies. A lot of beautiful poppies that would grow all the way around our area where I lived, which was a mile at the time. We hardly have that any more ... There was a thing called agent orange ... I remember one time climbing there and I saw the planes. I remember them spraying something alongside the river.

##### Water Contamination

Water as a main cause of cancer was described by 2 (50%) participants with asbestos in the water and the pipelines themselves perceived as cancer-causing:

To me I think it’s drinking water from the faucet ... Way back in the years there were cancer causing pipelines that were installed. And this person told my Dad that you won’t be surprised when people from the reservation will be experiencing this cancer.

Asbestos was sprayed and caused contamination of our water. I’m sure, I mean those run right through the areas where they have mined and it seeps into our water system.

#### Personal Choices as Risk Factors or Prevention: The Power of Food, Exercise, and Lifestyle

The personal choices participants linked to cancer risk or prevention had a recurring connection to food, exercise, and lifestyle. Diet choices were believed to be a main cause of cancer by 2 (50%) participants, and 2 (50%) expressed that substance-free living is important to cancer risk reduction.

Nutrition is prevention.

Exercise regularly. Don’t smoke. Don’t drink. Keep down on your fatty foods.

I think it helps prevent it. Because a strong body kind of resists it, I believe.

Sugar and processed foods were specifically mentioned as impacting cancer:

I truly believe that the sugar we take in has a lot to do with it ... Processed food is a part of it. If we sit around and do nothing, that unhealthy food builds up in our body. However, if we exercise, it is burned off, then the food nourishes our body.

However, one participant did not believe food and physical activity impacted the risk of cancer based on her personal experience of self-perceived healthy diet and activity but a diagnosis of cancer:

I guess I have to say no from my own experience, because I did that, you know, I exercised like crazy. I ate well. I made sure everything—I grew up in a home where we had our own garden and made sure that we ate healthy food, and that’s how I grew up.

#### Barriers

##### Distance and Transportation

Distance and transportation were cited as barriers to healthy lifestyle behaviors. There were 2 (50%) participants who described how lack of transportation impacted their ability to eat healthier and potentially participate in programming. There were 3 (75%) participants who described the distance to a grocery store with affordable prices and fresh options as barriers to healthy eating.

Having the transportation probably because right now my ride is down and all of us have a hard time making our way there.

You know that this [name of grocery store redacted] could have more healthy food and not so much processed food. Prepackaged stuff. If they could make other things available out there. I hardly ever go to [redacted] except to get immediate things, you know ... Their stuff is very high compared to others but can’t make the distance to [other location] every day.

##### The Built Environment

Participants described how the hills on their reservation and the busy road add challenges to their ability to exercise outdoors.

It’s dangerous to even cross the street. When I do go out, I usually go up with someone early in the morning.

Having the time and a level place to walk ... it is hilly where I live.

#### Acceptability of Diet and Physical Activity Programs

##### Overview

The participants were asked about a theoretical diet and physical activity program in their community, and all stated they would participate. When asked if they would participate when first diagnosed with cancer, all said yes with half providing no stipulation and half expressing concern about it interfering with treatment or the side effects of treatment being prohibitive.

Oh, yes, I sure would. I would love that.

Yeah, as long as it did it not interfere with my treatment.

When asked if their participation would have been influenced if it were explained that participating during cancer treatment could potentially improve cancer outcomes, all responded with strong affirmation, including those who had initial apprehension about it interfering with treatment.

##### Considerations for Program Development

The participants were invited to brainstorm the type of diet and physical activity program (prehab) they would want for their community. There were recurring responses about the need for support and encouragement, a central location, and education. Participants emphasized the need for peer support and encouragement, particularly the importance of including a cancer survivor as part of the program team.

People who have experienced cancer might be someone to have there. They know their issues and what they went through and can share ... Like a group where people could learn what’s going on with them and know somebody is listening to them that knows what is going to happen ... I think I was lost when I was told about having cancer, and it was scary in ways, and nobody ever told me you’re not going to die from this one.

All participants referenced their community center as the preferred location for prehab. While there was consistency on the community center as the chosen central location, upon further probing, it became apparent that each district of the reservation has its own community center, and while the participants were uniform on the category of “community center,” they were referencing different candidate buildings. Reflective of the concern that lack of transportation is a barrier to a healthy lifestyle, one participant recommended that transportation to the central location be provided by the program. Participants also voiced interest in education, delivered either by peers or health professionals.

Somebody in oncology who can share what happens as nobody gets taught about these things. I was having to get on the computer to find out what this word means and the different services available. It’d be nice if someone had sat me down and kind of counseled me on it and what could happen.

First you have to educate them on the disease and what it does to their body. Then you have to cover the things that could be promoting it to happen. And lastly the things that could change that.

There was no clear consensus on how often the program should meet. One participant requested a daily program. The others recommended 1-3 sessions weekly. There was no consensus on how long each session should occur with a range of responses from 30 to 120 minutes, but 3 (75%) supported 1 hour as appropriate. Participants practiced a wide range of physical activities including treadmill, weights, biking, running, gardening, gathering acorns, and aerobics. All 4 mentioned walking as a favorable regular activity. There were 3 (75%) participants who described going to a fitness center as favorable. The participants were presented with a list of food items that have been included in other prehab programs and asked about their likelihood of consuming these if the food items were provided. On a scale of 1-5 with 1=absolutely would not eat and 5=absolutely would eat, the participants viewed fiber powder and flax seeds negatively, protein shakes neutrally, and walnuts as highly favorable, with all 4 giving a score of 4-5 likelihood of consuming walnuts as part of a prehab program.

#### Acceptability of Monitoring and Biospecimen Collection

All participants responded positively to the following forms of monitoring: measurement of heart rate, blood pressure, and weight; blood collection to measure glucose, cholesterol, and levels of inflammation; and biospecimen collection (ie, tumor tissue) to measure inflammation. Half brought up that this is a personal choice, and they were not sure if others would agree to monitoring.

I would. I don’t know about other people but I can. It’s a necessary part of what you do.

Yeah. It’s important to be monitored when you’ve got cancer.

The question about biospecimen collection generated more discussion. It was first explained that measuring inflammation in the cancer tissue before and after a prehab program is one way to see if the program is effective, and that it can be done without any extra procedures beyond those required for standard cancer treatment. Then the participants were asked, “Looking back, do you think you would be comfortable with your biopsy tissue and the tumor removed by the surgeon to be analyzed for inflammation to see if a diet and physical activity program can change cancer cells?” All said they would with 3 of the participants providing a yes without stipulation, and 1 person asking for further clarification. After repeating the explanation, the individual responded, “I definitely agree with that.”

## Discussion

### Principal Findings

To our knowledge, this is the first study to engage San Carlos Apache cancer survivors for their perspectives on lifestyle modifications in diet and physical activity and the cancer journey. It is the investigators’ long-term goal to work with the tribe and health facility to collaboratively develop a nutrition and physical activity prehab program that serves patients with cancer. Prior to programmatic development, the team recognized that it was critical to invite community members into the discussion early as a first phase of research to learn if such programming would be appealing and what background the community prioritizes. From these interviews, important themes and subthemes were highlighted: (1) cancer is perceived as a serious health condition in the community; (2) occupational exposures, chemical exposures, and water contamination on the reservation are perceived as cancer-causing threats; (3) healthy foods, regular exercise, and avoiding harmful substances are mostly perceived as measures an individual can use to try to prevent cancer, though there is recognition that it is not a guaranteed protection; (4) lack of transportation, distance to affordable groceries and recreational centers, and hilly and high-traffic roads are barriers to healthy habits for this community; (5) there is high interest in a prehab program geared toward patients with cancer that should include components of encouragement and education and take place at a central location; and (6) standard monitoring practiced in published prehab programs, which includes blood and tumor-tissue samples, is acceptable to the individual cancer survivors who participated in this study with the important point made that it is their personal view, and openly sharing a clear rationale may encourage wider acceptability.

### Comparison to Prior Work

Lifestyle interventions for optimizing cancer outcomes are undergoing a paradigm shift in which earlier engagement (ie, prehab) holds promise for greater impact, including modulating inflammation. Obesity is considered a pro-inflammatory condition, and the synergy between obesity and inflammation is thought to influence cancer risk and progression. With an increased risk of obesity, American Indian/Alaska Native populations carry an increased risk of obesity-related cancers and worse outcomes [24-26]. Previous studies with participants from the general population have shown that prehab interventions have modulated inflammation in multiple cancer subtypes as measured by biomarkers in windows as brief as 3-5 weeks between diagnosis and operation [32-36]. The preoperative period is a unique window before treatment side effects have set in and might be more conducive to patient participation while additionally fostering healthy behaviors for treatment and survivorship ahead. Such studies have never been implemented for American Indian/Alaska Native populations. With a disproportionate burden of obesity and cancer mortality, prehab interventions during this diagnosis-to-surgery window of opportunity could provide gains in health equity for American Indian/Alaska Native populations, a group underrepresented in clinical research [37].

Leaders in the field have published their recommendation that well-designed interventions should include tumor-derived tissue from diagnostic biopsies and surgical resections to evaluate the effects of the intervention on the tumor microenvironment [12,38]. While this is an accepted measure with general populations, it is a delicate subject with American Indian/Alaska Native audiences, as some tribes have experienced breaches in the ethical conduct of research and misuse of their biospecimens [39]. Our team wanted to proactively address this component of prehab trials to understand whether this would be an acceptable method of measuring outcomes with San Carlos Apache participants. Prepared for apprehension, we were encouraged to learn that the participants saw the benefit of monitoring vitals, blood samples, and tissue samples. We hold this trust in the highest regard and comply with the University of Arizona’s rigorous process for tribal-related research (ABOR1-118 Tribal Consultation Policy), which recognizes the fundamental principles of tribal sovereignty and requires documented evidence of consultation and approval when conducting research with Native Nations [40]. These processes and special considerations are important to highlight for any team interested in research with a tribal partner, and prospective investigators should bear in mind that each individual tribe determines the process that must be followed at all steps of the investigation.

Dietary interventions rich in omega-fatty acids have been conducted outside the prehab context to alter inflammation in various disease conditions including cancer. These studies have featured omega-rich foods, like walnuts, which have a high content of polyunsaturated fatty acids and antioxidants that can improve blood lipid profiles, reduce oxidative stress, influence inflammatory biomarkers, and reduce tumor growth, offering a natural and tasty way of improving outcomes [41-54]. This single food intervention affords simplicity in delivery, has previously demonstrated high uptake and adherence, and was highly endorsed by the San Carlos Apache participants. One person commented that walnuts grow wild on the reservation and would be perceived as a more natural, traditional food option.

### Strengths and Limitations

This study has limitations. With 4 participants, the findings may not be fully representative of all San Carlos Apache patients with cancer; however, the participants were consistent in most of their responses, which may have achieved saturation or sufficiency of themes. Qualitative researchers are recognizing that sufficiency depends on the rigor of the process and the richness of the data it generates, more than the absolute number of participants [55]. With the length of time these participants invested in the interviews and the candor of their responses, we believe this to be a strong and meaningful data set. Another limitation is that cancer survivors rather than patients who were newly diagnosed with cancer were interviewed. Survivors were selected because of their line of sight on the full spectrum of the cancer experience in their community, which had the advantage of providing reflections outside the acute exhaustion of a new diagnosis. This has the potential for recall bias and might not have rendered their real-time experience as accurately. Finally, a surprising result was the very high level of vigorous or moderate physical activity the participants reported, which is more active than average prehab patients and should be explored further.

### Future Directions

Based on these qualitative data, San Carlos Apache members have demonstrated interest in a nutrition and exercise program delivered between diagnosis and oncologic surgery that includes encouragement, education, standard monitoring methods, and support in overcoming barriers to participation. The participants offered valuable insight on the specifics that would adapt published prehab models to their community. Such an intervention would attempt biochemical change using food as medicine and exercise as resilience, which is reflective of the wider momentum that American Indian/Alaska Native scholars are building in national discussions on the topics of Indigenous food sovereignty, decolonizing the diet, embracing traditional perspectives, and restoring balance [56-59].

### Conclusions

American Indian/Alaska Native populations are known to be underrepresented in clinical trials and research, and even more importantly, they have never been included in the adaptation and participation of an intervention designed to modulate inflammation through diet and exercise prior to cancer surgery. San Carlos Apache would be the first tribal partner to conduct such a program and deliver the emerging science of prehab to a population that has yet to be included in this field of work. Future directions should include the findings from these interviews with continued tribal collaboration to develop a program specific to the San Carlos Apache community.

## Abbreviations

IPAQ: International Physical Activity Questionnaire
REAPS: Rapid Eating Assessment for Participants-Shortened Version
SCAHC: San Carlos Apache Healthcare Corporation
